# Analysis of the conformational heterogeneity of the Rieske iron–sulfur protein in complex III_2_ by cryo-EM

**DOI:** 10.1107/S2052252522010570

**Published:** 2023-01-01

**Authors:** Jan-Philip Wieferig, Werner Kühlbrandt

**Affiliations:** a Department of Structural Biology, Max Planck Institute of Biophysics, Max-von-Laue-Strasse 3, 60438 Frankfurt am Main, Germany; Max Planck Institute of Molecular Physiology, Germany

**Keywords:** conformational heterogeneity, complex III_2_, Rieske domains, iron–sulfur proteins

## Abstract

Movement of the Rieske domain of the iron–sulfur protein is essential for intramolecular electron transfer within complex III_2_ (CIII_2_) of the respiratory chain as it bridges a gap in the cofactor chain towards the electron acceptor cytochrome *c*. We present cryo-EM structures of CIII_2_ from *Yarrowia lipolytica* at resolutions up to 2.0 Å under different conditions, with different redox states of the cofactors of the high-potential chain. All possible permutations of three primary positions were observed, indicating that the two halves of the dimeric complex act independently. Addition of the substrate analogue decylubiquinone to CIII_2_ with a reduced high-potential chain increased the occupancy of the Q_o_ site. The extent of Rieske domain interactions through hydrogen bonds to the cytochrome *b* and cytochrome *c*_1_ subunits varied depending on the redox state and substrate. In the absence of quinols, the reduced Rieske domain interacted more closely with cytochrome *b* and cytochrome *c*_1_ than in the oxidized state. Upon addition of the inhibitor antimycin A, the heterogeneity of the *cd*_1_-helix and *ef*-loop increased, which may be indicative of a long-range effect on the Rieske domain.

## Abstract 2

Cryo-EM structures of complex III_2_ under a range of redox conditions with or without exogenous substrates provide evidence for redox- and substrate-dependent interaction of the Rieske iron–sulfur protein with quinone in the Q_o_ site and the cytochrome *b* and cytochrome *c*_1_ subunits.

## 1. Introduction

The cytochrome *bc*_1_ complex, also known as respiratory complex III (CIII), in the electron-transfer chain of the inner mitochondrial membrane is a homodimer (CIII_2_) of two biochemically identical protomers. Each protomer consists of three subunits that form the catalytic core of the complex, plus up to eight supernumerary subunits. CIII_2_ catalyses the transfer of electrons from the lipophilic two-electron donor ubiquinol (QH_2_) to the water-soluble, one-electron acceptor protein cytochrome *c*, contributing to an electrochemical proton gradient across the membrane. An ATP synthase then uses the gradient to generate ATP. Electron transfer within the complex occurs through the prosthetic groups of the three catalytic core subunits: heme *b*_L_ and heme *b*_H_ of cytochrome *b* (cyt *b*), heme *c*_1_ of cytochrome *c*_1_ (cyt *c*_1_) and the [2Fe–2S] cluster of the iron–sulfur protein (ISP), often called the Rieske protein after its discoverer (Rieske *et al.*, 1964 ▸). The Rieske iron–sulfur protein also participates in electron transport in the closely related cytochrome *b*_6_ *f* complex from photosynthetic membranes (Kurisu *et al.*, 2003 ▸; Malone *et al.*, 2019 ▸). Translocation of protons across the membrane takes place through QH_2_, which is oxidized by CIII_2_ according to the modified Q-cycle mechanism (Mitchell, 1975 ▸; Crofts *et al.*, 1983 ▸). For every molecule of QH_2_ that is oxidized to ubiquinone (Q), two protons are released into the intermembrane space and one proton is taken up from the matrix. In the Q-cycle mechanism, proton release results from the oxidation of QH_2_ at the Q_o_ site. From the Q_o_ site, one electron is transferred through the high-potential chain that consists of a [2Fe–2S] cluster and heme *c*_1_ to cytochrome *c*. The second electron is transferred through the two *b*-type hemes, which constitute the low-potential chain, to Q bound at the Q_i_ site, a second Q/QH_2_ binding site. The intermediate semiquinone (SQ) radical generated at the Q_i_ site is stabilized and then fully reduced to QH_2_ upon the oxidation of another QH_2_ at the Q_o_ site in a second turnover of the cycle (Sousa *et al.*, 2018 ▸). The bifurcation of electrons originating from QH_2_ that is oxidized at the Q_o_ site is therefore crucial for energy conservation and charge separation to generate the proton-motive force (Mitchell, 1976 ▸) in bacteria and mitochondria.

CIII_2_ is well-characterized and a wealth of information about its structure and mechanism has accumulated over the years (for reviews, see Sarewicz *et al.*, 2021 ▸; Crofts, 2021 ▸; Crofts *et al.*, 2017 ▸; Sarewicz & Osyczka, 2015 ▸; Berry *et al.*, 2013 ▸). Nevertheless, some important questions about the reaction mechanisms at the Q_o_ site remain unanswered. These reactions ensure electron bifurcation, optimize the production of reactive oxygen species (ROS; Borek *et al.*, 2008 ▸; Pagacz *et al.*, 2021 ▸) and minimize short-circuit reactions to ensure high efficiency of energy conversion (reviewed in Sarewicz *et al.*, 2021 ▸). To date, structural information on the quinone in the Q_o_ site is limited, as no structures of defined catalytic states are available (Kao & Hunte, 2022 ▸).

From the initial crystal structures of cytochrome CIII_2_ (Xia *et al.*, 1997 ▸; Zhang *et al.*, 1998 ▸; Kim *et al.*, 1998 ▸) and subsequent mutational studies, it is clear that the mobile domain of the Rieske protein must relocate within the complex so that the [2Fe–2S] cluster at the tip of the solvent-exposed ISP domain (Rieske domain) can shuttle electrons across the energetic gap in the high-potential chain between the putative QH_2_ binding at the Q_o_ site and heme *c*_1_. The Rieske domain is tethered to the complex by one transmembrane helix and a connecting flexible linker that acts as a hinge. The [2Fe–2S] cluster can thus be positioned close to the Q_o_ site (referred to here as the *b* or proximal position), which is formed by cyt *b*. Alternatively, the Fe–S cluster is positioned closer to cyt *c*_1_ (the *c* or distal position). In intermediate positions, the tip of the Rieske domain is close to the *ef*-loop of cyt *b* (reviewed by Darrouzet *et al.*, 2001 ▸).

Electron paramagnetic resonance (EPR) studies revealed redox-dependent shifts in the equilibrium of positions that are occupied by the Rieske domain (Brugna *et al.*, 2000 ▸). These shifts were thought to be linked to the formation of a hydrogen bond between the redox-sensitive cluster-ligating histidine and either the substrate or product in the Q_o_ site. Reduction of the [2Fe–2S] cluster favours a hydrogen bond between the histidine and Q, whereas the oxidized cluster would favour a hydrogen bond to QH_2_ (Crofts *et al.*, 1999 ▸). X-ray structures of CIII_2_ indicate that the position of the Rieske domain depends on both the Q_o_-site occupation and on concomitant structural changes on the surface of cyt *b*. Therefore, cyt *b* was proposed to act as a catalytic switch for the capture and release of the Rieske domain (Esser *et al.*, 2006 ▸). Moreover, the Q_i_-site inhibitor antimycin A has been implicated in affecting the mobility of the Rieske domain by favouring its detachment from cyt *b*, although the exact effects appear to be less clear (Sarewicz *et al.*, 2009 ▸).

The above findings support a gating function of the Rieske domain for the reaction at the Q_o_ site with possible involvement of cyt *b*. However, the lack of structural information on the binding of Q/QH_2_ and difficulties in detecting SQ intermediates in the Q_o_ site, combined with the complexities of electron bifurcation or confurcation at this site, have led to multiple hypotheses for possible gating mechanisms (see the review by Sarewicz *et al.*, 2021 ▸). For example, the so-called ‘double-gating’ mechanisms (Osyczka *et al.*, 2004 ▸, 2005 ▸) or ‘logic gating’ (Rich, 2004 ▸) would minimize short circuits while ensuring reversible reactions. In these mechanisms, the redox states of both the [2Fe–2S] cluster and heme *b*_L_ control the interactions of the Q_o_ site with Q/QH_2_. If the two-electron redox reactions of QH_2_/Q at the Q_o_ site were concerted rather than sequential, bypassing reactions would be prevented and the formation of a highly reactive SQ intermediate would be avoided. To date, however, there is no single mechanism that explains all experimental observations (Sarewicz *et al.*, 2021 ▸).

In crystallographic studies, problems of domain mobility can be overcome by P_f_-type inhibitors, which fix the Rieske domain in the *b* position where its cluster-bearing tip is tightly docked to the surface of cyt *b*. A second class of Q_o_-site inhibitors, the P_m_­-type inhibitors, prevent attachment of the Rieske domain to cyt *b*. In some crystal forms these inhibitors stabilize intermediate or *c* positions in which the tip of the Rieske domain is detached from the docking crater and is closer to heme *c*_1_ (Esser *et al.*, 2004 ▸; Berry *et al.*, 2013 ▸). One advantage of cryo-EM over crystallography is that it can analyse conformational heterogeneity in a population of complexes that are simultaneously present on the EM grid (Scheres, 2016 ▸; Nakane *et al.*, 2018 ▸; Punjani & Fleet, 2021 ▸). It should therefore be possible to resolve different positions of the Rieske domain within a single sample of CIII_2_. This was recently demonstrated for CIII_2_ from plants using 3D variability analysis. In this case, a coordinated antiparallel motion of the Rieske domain protomers was found (Maldonado *et al.*, 2021 ▸). With bacterial cytochrome *bc*_1_, which only contains the catalytic core subunits, 3D classification was used to separate conformationally asymmetric dimers (antiparallel positions of the Rieske domain protomers) as well as two symmetric dimers (Steimle *et al.*, 2021 ▸). In yeast CIII_2_, the Rieske domain movements in the two CIII protomers did not appear to be coordinated (Di Trani *et al.*, 2022 ▸).

Our aim was to elucidate the structure of CIII_2_ from the yeast *Yarrowia lipolytica* by cryo-EM and to investigate the mobility of the small, ∼14 kDa Rieske domain under different redox conditions in the absence or presence of substrate analogues and inhibitors. We determined the structure of CIII_2_ bound to the P_f_-type inhibitor atovaquone and the Q_i_-site inhibitor antimycin A, as well as structures of CIII_2_ with a reduced or oxidized high-potential chain, with and without the added substrate analogues decylubiquinone (DQ) or decyl­ubiquinol (DQH_2_). The enzyme–product complex was expected to form when the reduced Rieske domain is in the *b* position and quinone occupies the Q_o_ site. Focused 3D classifications improved the density for the mobile Rieske domain, which allowed us to resolve several different positions in a conformationally heterogeneous sample as well as distinct changes in its position depending on experimental conditions.

## 2. Methods

### 2.1. Purification of CIII_2_

CIII_2_ was purified from detergent-solubilized mitochondrial membrane preparations after depletion of the His-tagged respiratory chain complex I by immobilized ion-affinity chromatography, as described by Parey *et al.* (2021 ▸).

In a protocol adapted from Pálsdóttir & Hunte (2003 ▸), CIII_2_ was purified by ion-exchange chromatography followed by size-exclusion chromatography using Poros GoPure HQ50 and Superdex 200 Increase columns. The sample was diluted in 50 m*M* Tris–HCl buffer with 350 m*M* NaCl, 1 m*M* EDTA pH 8.0 and 0.025% DDM pH 8.0 prior to running a 350–1000 m*M* NaCl gradient. Size-exclusion chromatography was performed at reduced buffer (20 m*M*) and salt (40 m*M*) concentrations.

### 2.2. Cryo-EM specimen preparation and data acquisition

CIII_2_ was concentrated to ∼5 mg ml^−1^ (∼10 µ*M*) in 20 m*M* Tris–HCl pH 8.0, 40 m*M* NaCl, 1 m*M* EDTA pH 8.0, 0.025% *n*-dodecyl-β-d-maltoside (DDM) . Potassium hexaferricyanide was added to 500 µ*M* (sample +FCN) and sodium ascorbate to 25 m*M* (sample +NaAsc). Atovaquone (Sigma, catalogue No. A7986) was added at 120 µ*M* together with antimycin A (Sigma, catalogue No. A8674) at 125 µ*M* (sample +atovaquone+antimycin A). Decylubiquinol was added at 1 m*M* with or without 188 µ*M* antimycin A (samples +DQH2 and +DQH2+antimycin A). Decylubiquinone (Sigma, catalogue No. D7911) was added at 330 µ*M* with or without 25 m*M* sodium ascorbate (samples +DQ and +DQ+NaAsc). Hydrophobic ligands were incubated with the sample for at least 30 min on ice before vitrification. Decylubiquinone was reduced with sodium borohydride to obtain decylubiquinol (Graham & Rickwood, 1997 ▸).

3 µl of sample was applied onto glow-discharged Quantifoil R2/2 copper–carbon grids or UltrAuFoil R1.2/1.3 gold grids and blotted for 5–9 s with a Mark IV Vitrobot at 10°C and 100% relative humidity.

UV–Vis spectra of CIII_2_ preparations were recorded using a Varian Cary 50 with the same CIII_2_:additive ratio as for cryo-EM samples.

Images were acquired using a Titan Krios (ThermoFisher Scientific) equipped with a K3 camera (Gatan) in counting mode and a Gatan BioQuantum energy filter with a slit width of 20 eV. The fluence was set to ∼1.1 e Å^−2^ per frame and 50-frame movie stacks were recorded in 2.7 s exposures at a nominal magnification of 105k× with a resulting pixel size of 0.83 Å. Defocus values were set in the range −0.9 to −2.3 µm.

### 2.3. Image processing and modelling

Micrographs were processed in *RELION*-3.1 (Zivanov *et al.*, 2018 ▸). The *RELION* implementation of *MotionCor*2 (Zheng *et al.*, 2017 ▸) was used for drift correction. *Gctf* (Zhang, 2016 ▸) was used for the initial CTF estimation. The template-based autopicker was used and 3D classifications (*C*1) were performed to remove bad picks before 3D autorefinement (*C*1). The resolution was improved by two iterations of CTF refinement, one before and one after per-particle drift correction and dose-weighting with Bayesian polishing. Polished particles were subjected to a final consensus 3D autorefinement (*C*1) that yielded high-resolution maps of CIII_2_ with blurred Rieske domains.

Particles were *C*2 symmetry-expanded (*relion_particle_symmetry_expand*) and signal outside a mask around the Rieske domain was removed (particle subtraction) for focused 3D classifications. Classifications were performed with six classes without alignment, with the mask used for particle subtraction as a reference mask, a 15 Å low-pass filtered consensus refinement map as a reference and a *T* value of 256. The particle subtraction was reverted and separate 3D autorefinements (*C*1) were performed with local searches and without a reference mask for selected classes.

To resolve both Rieske domains of CIII_2_, particles were separated according to the classes of both the particle and its symmetry-expanded duplicate rotated by 180° prior to 3D autorefinement.

*C*2 symmetry was applied to symmetric dimers and the map resulting from the 2.0 Å consensus refinement of combined data sets. Local resolution maps were calculated in *RELION*-3.1.

*Phyre* ^2^ (Kelley *et al.*, 2015 ▸) was used to build initial homology models. These models were then refined with *Coot* (Emsley *et al.*, 2010 ▸) and *REFMAC*5 (Murshudov *et al.*, 2011 ▸). Figures showing atomic models and maps were created with *ChimeraX* (Pettersen *et al.*, 2021 ▸). Water molecules were modelled with *phenix.douse* (Afonine *et al.*, 2012 ▸)*.*

## 3. Results

CIII_2_ from *Y. lipolytica* was solubilized and purified with DDM at pH 8.0 under aerobic conditions. UV–Vis spectrophotometry showed that the *b*-type hemes were oxidized but the high-potential *c*-type hemes remained partially (∼40%) reduced after purification (Supplementary Fig. S1). In addition to this apo state without additives, we prepared samples to investigate potential effects on the position of the Rieske domain. Additives included commonly used reductants or oxidants of the high-potential chain (sodium ascorbate and potassium ferricyanide), Q_o_- and Q_i_-site inhibitors (atovaquone and antimycin A), substrate and product analogues (DQH_2_ and DQ) and combinations thereof. The cryo-EM structure of CIII_2_ with bound atovaquone served as a reference for the complex with an immobilized Rieske domain, in addition to the corresponding X-ray structure (Birth *et al.* (2014 ▸).

A total of nine CIII_2_ samples were prepared for cryo-EM.

(i) Apo: without additives (partially reduced).
(ii) +Antimycin A: with antimycin A only.
(iii) +NaAsc: reduced with ascorbate.
(iv) +FCN: oxidized with potassium ferricyanide.
(v) +Atovaquone+antimycin A: with the P_f_-type inhibitor atovaquone and the Q_i_-site inhibitor antimycin A.
(vi) +DQH2: with the substrate analogue decylubiquinol.
(vii) +DQH2+antimycin A: with DQH_2_ and antimycin A.
(viii) +DQ: with decylubiquinone (high-potential chain oxidized).
(ix) +DQ+NaAsc: reduced by ascorbate and DQ.

**Figure 1:**
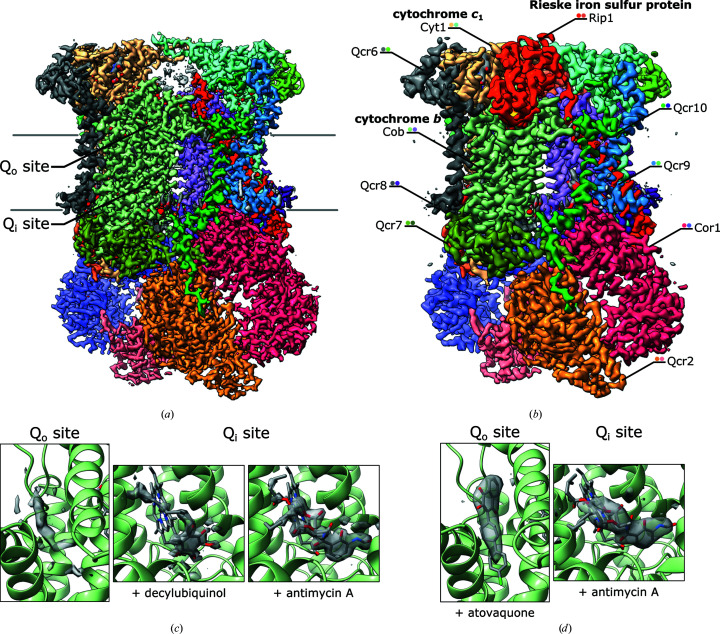
Cryo-EM density maps of respiratory complex III from *Y. lipolytica* at 2.0 Å resolution (*a*) (combined data sets; the position of the lipid bilayer is indicated by grey lines) and 3.3 Å resolution (*b*) (+atovaquone+antimycin A) with the three subunits Cyt1, Rip1 and Cob that form the catalytic core and all seven supernumerary subunits found in yeasts. The solvent-exposed domain of the Rieske iron–sulfur protein was not resolved in the consensus refinement of all data sets of samples without added inhibitor (*a*), whereas it was fixed in the *b* position (red, with the [2Fe–2S] cluster in yellow and orange) by the P_f_-type inhibitor atovaquone (*b*). The lower panels show ligand densities after the refinement of individual data sets (*c*, *d*). After its addition, decylubiquinol was resolved in the Q_i_ site, while density in the Q_o_ site remained poorly defined. The inhibitor antimycin A displaced decylubiquinol from the Q_i_ site when both were added (*c*). Atovaquone and antimycin A were resolved in the Q_o_ and Q_i_ sites, respectively, in the 3.3 Å resoution map (*d*). All maps were sharpened by a *B* factor of −30 Å^2^.

The overall resolution of the consensus cryo-EM maps ranged from 2.1 to 3.3 Å for all samples. The consensus 3D refinement of combined particle images from all samples except the sample with atovaquone yielded a map with a resolution of 2.0 Å [Fig. 1 ▸(*a*)]. The three subunits of the catalytic core Cob (cyt *b*), Cyt1 (cyt *c*_1_) and the transmembrane domain of Rip1 (ISP), as well as the seven supernumerary subunits of yeast CIII, Cor1, Qcr2, Qcr6–9 and the less tightly bound Qcr10, were well-resolved, consistent with other complete structures of CIII_2_ within respiratory supercomplexes from *Saccharomyces cerevisiae* (Rathore *et al.*, 2019 ▸; Hartley *et al.*, 2019 ▸, 2020 ▸). Density for the hydrophilic Rieske domains were equally poor in *C*1 and *C*2 refinements in all samples except that with atovaquone. Ten cardiolipin molecules on the matrix side, four molecules of DDM, four phosphocholines, four phosphatidylethanolamines and two phosphatidic acid molecules were modelled with truncated fatty-acid chains. We identified 1326 water molecules in the 2 Å resolution map of the dimeric complex.

Except for the sample with added P_f_-type inhibitor, the Rieske domain map density was weak, as is commonly observed in uninhibited and hence unrestrained CIII_2_. In the consensus refinements of the apo state, rod-shaped densities were observed in the Q_o_ and Q_i_ sites at low map thresholds, which could not be reliably assigned to particular ligands. We conclude that most of the endogenous quinone was lost during purification.

In the consensus refinements of the +atovaquone+antimycin A, +DQH2+antimycin A and +DQH2 samples, the Q_i_ site was occupied by antimycin A or DQH_2_ [Figs. 1 ▸(*c*) and 1 ▸(*d*)]. The poor solubility of DQ limited the accessible concentration range, which may explain why we did not observe density in the Q_i_ site in the consensus refinements. Atovaquone was resolved in the Q_o_ site in the +atovaquone+antimycin A sample, with the Rieske domain fixed in the *b* position [Fig. 1 ▸(*d*)]. In samples with added DQH_2_ or DQ, the densities in the Q_o_ sites increased slightly but the density for the Rieske domain remained weak [Fig. 1 ▸(*c*)].

### 3.1. Focused 3D classifications and local 3D refinements

Focused 3D classification (Bai *et al.*, 2015 ▸) was performed on all data sets in order to sort the images of the CIII_2_ particles according to the positions of the two ∼14 kDa small Rieske domains. Firstly, particles after 3D refinement without imposed symmetry were *C*2 symmetry-expanded to allow the classification of both Rieske domains on one side of the complex. Then, all signal from particle images outside a small mask that encompassed the volume of the Rieske domain in the *b*, intermediate and *c* positions was removed by particle subtraction in *RELION*. This mask included some residues of cyt *c*_1_ and cyt *b* that line the cavity in which the Rieske domain moves, but it was designed to exclude as much of the other subunits as possible. Otherwise, classifications would be driven by these less flexible regions rather than by the Rieske domain. 3D classifications were then performed with particle orientations fixed as determined in the prior consensus refinements that had yielded high-resolution maps of CIII_2_ with poorly resolved Rieske domains (Supplementary Fig. S2). Classification improved the density of the Rieske domain, which was seen to assume one of three different positions (Supplementary Fig. S3).

**Figure 2:**
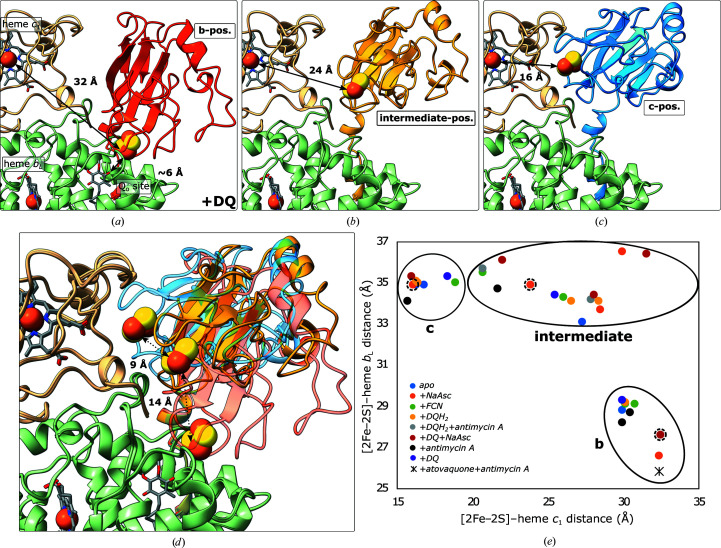
Typical *b* (red), intermediate (yellow) and *c* (blue) positions of the Rieske domain after focused 3D classification and refinement (*a*, *b*, *c*) and a superposition of the three models (*d*). The range of different positions of the Rieske domain in all samples is visualized in the plot of the distances between Fe2 in the [2Fe–2S] cluster and Fe of heme *b*_L_ and heme *c*_1_, respectively (*e*). The three positions in (*a*)–(*d*) are marked by dashed circles in (*e*).

Classes were then subjected to separate local 3D refinements after reversing the particle subtraction without imposing symmetry. Density for one of the Rieske domains improved and different positions were resolved, indicating a narrower distribution of occupied positions within the continuum of possible orientations. Distances between Fe2 of the [2Fe–2S] cluster and Fe of heme *b*_L_ and heme *c*_1_ were measured and plotted to visualize the range of positions (Fig. 2 ▸). The density of the second Rieske domain was blurred in these maps, as expected (Steimle *et al.*, 2021 ▸; Di Trani *et al.*, 2022 ▸).

### 3.2. The *b* position

**Figure 3:**
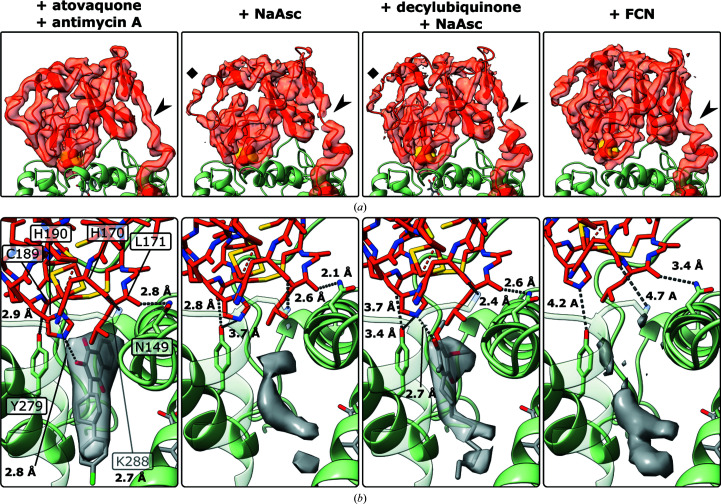
Rieske domain (red) in the *b* position. (*a*) shows the cryo-EM density of the ISP. The hinge (black arrowhead) extended when atovaquone was bound (Darrouzet *et al.*, 2000 ▸; Birth *et al.*, 2014 ▸). When the high-potential chain was reduced by ascorbate, density at the hinge and the surface (black square) of the Rieske domain was weak, indicating local heterogeneity despite strong density for the [2Fe–2S] cluster (yellow) and the surrounding tip region. When the high-potential chain was oxidized with ferricyanide, the hinge rigidified and turned into an α-helix. Details of the docking crater, which is formed by cyt *b* (light green), are depicted in (*b*) with density for the Q_o_ site. Atovaquone induced tight docking of the Rieske domain, as shown by strong density for the Rieske domain in (*a*) and close interaction of the tip of the Rieske domain with the docking crater and atovaquone in (*b*). When CIII_2_ was reduced with ascorbate, the Rieske domain was within hydrogen-bonding distance of cyt *b* and potential Q_o_-site occupants. Density in the Q_o_ site, in a similar position to atovaquone, increased when decylubiquinone was added together with ascorbate. The Rieske domain was beyond hydrogen-bonding distance to cyt *b* in the *b* positions that were observed with an oxidized high-potential chain (+FCN) and also the other tested conditions (Supplementary Fig. S4). For better visualization, a positive *B* factor of +10 Å^2^ was applied to limit the map resolution. Exemplary maps coloured by local resolution are shown in Supplementary Fig. S5.

A closer look indicated subtle differences in the position of the Rieske domain and the occupancy of the Q_o_ site (Fig. 3 ▸). In all of these *b* positions the distance between the [2Fe–2S] cluster and the Q_o_ site (using the atovaquone binding site as a reference) was less than ∼10 Å. The cluster-binding tip of the Rieske domain was well-resolved and density for the [2Fe–2S] cluster was strong. Using the sample of CIII_2_ with an excess of DQH_2_, density for the Rieske domain in this position did not greatly improve (Supplementary Fig. S4) compared with the other samples.

Atovaquone induced very tight binding of the tip of the Rieske domain to cyt *b.* As expected for a P_f_-type inhibitor, the hinge region that tethers the mobile Rieske domain to the transmembrane domain was in an extended conformation (Darrouzet *et al.*, 2000 ▸). Together with the widening of the docking crater (Esser *et al.*, 2006 ▸), this allowed close association of the Rieske domain with cyt *b*. Atovaquone formed a hydrogen bond to the cluster-ligating His190 (2.8 Å). In addition, Cys189, His170 and Leu171 of the Rieske domain formed hydrogen bonds to Tyr279, Lys288 and Asn149 of cyt *b*, as previously observed (Birth *et al.*, 2014 ▸). These inter­actions stabilized the whole Rieske domain, improving the local resolution. The docking crater did not widen in samples without a P_f_-type inhibitor (Supplementary Fig. S6).

The Rieske domain approached the P_f_-type inhibitor-induced structure most closely when the high-potential chain was reduced by sodium ascorbate, with a similar hydrogen-bonding distance between the Rieske domain and cyt *b*. Density inside the Q_o_ site was weak, suggesting that no substrate had remained bound during sample preparation.

When DQ and ascorbate were added after purification (+DQ+NaAsc) density in the Q_o_ site increased, indicating a possible low occupancy of DQ in the Q_o_ site. The additional density was in a position similar to atovaquone. The Rieske domain was in a position in which Tyr279 and Cys189 were further apart (3.7 Å) and His190 was closer to Tyr279 (3.4 Å) compared with the ascorbate-reduced sample without added DQ. His190 was ∼2.7 Å away from the DQ density in the Q_o_ site.

The Fe–S cluster-bearing tip of the Rieske domain was well-resolved in the two ascorbate-reduced samples. Density at the hinge, where the transition from helix to random coil occurs with P_f_-type inhibitors bound, and density distal to the tip was weak, indicating heterogeneity in these regions. A shift towards the *b* position in the equilibrium of positions was not observed (Supplementary Fig. S3).

In all the other tested conditions with the high-potential chain partially reduced, oxidized or reduced by DQH_2_, the Rieske domain was more distal, beyond hydrogen-bonding distance to cyt *b*, and the hinge formed an α-helix. Density in the Q_o_ site was weak and more proximal compared with +DQ+NaAsc (Fig. 3 ▸, Supplementary Fig. S4). When antimycin A was added with DQH_2_, the Rieske domain did not occupy the *b* position.

### 3.3. The *c* and intermediate positions

**Figure 4:**
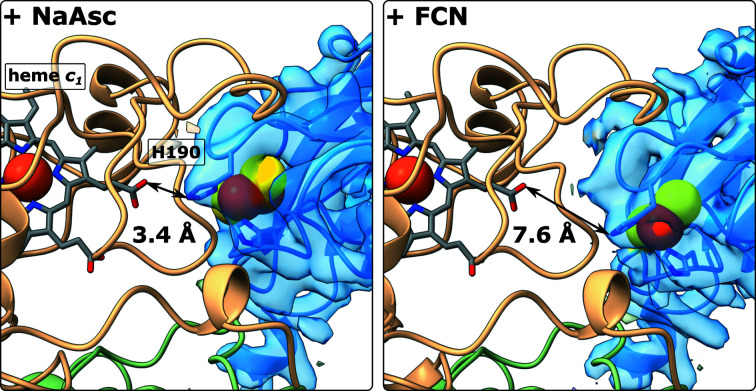
His190 ligating the Fe–S cluster was 3.4 and 7.6 Å away from the propionic acid group of heme *c*_1_ in the *c* positions of the Rieske domain (blue) in ascorbate-reduced (+NaAsc) and ferricyanide-oxidized (+FCN) CIII_2_, respectively.

**Figure 5:**
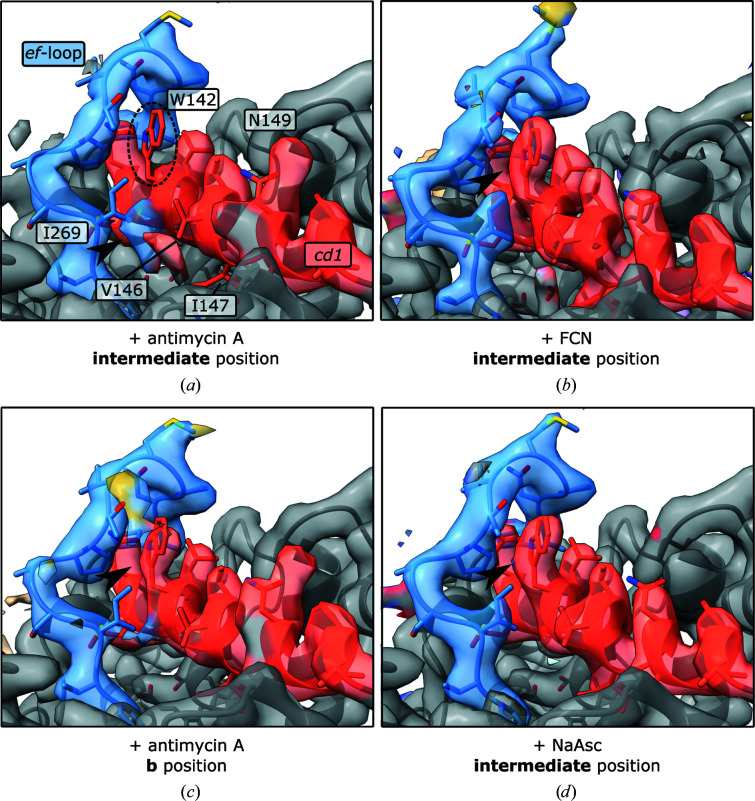
Cyt *b* in samples of CIII_2_ prepared with antimycin A (*a*, *c*), oxidized with ferricyanide (*b*) or reduced with ascorbate (*d*). (*a*) Density for Trp142 (dashed ellipse) of the *cd*_1_-helix and Ile269 of the *ef*-loop was missing in maps of CIII_2_ with only antimycin A added when the Rieske domain (cropped out, residual yellow density) was in the intermediate position. Additional density was visible at a position near the Q_o_ site on the left of the *cd*_1_-helix, partially overlapping with the position of Ile269 of the *ef*-loop (arrow). Densities for Val146, Ileu147, Cys148 and Asn149 were ambiguous as in the apo sample (Supplementary Fig. S7). (*c*) With the Rieske domain in the *b* position, density for Trp142 was resolved in the expected position beneath the *ef*-loop, as in samples with added ferricyanide (*b*), ascorbate (*d*), DQH_2_ or DQ. All side chains of the *ef*-loop and *cd*_1_-helix were also resolved with the Rieske domain in the intermediate position. The overall resolutions of the maps were 3.0 Å (*a*), 3.0 Å (*c*), 2.3 Å (*b*) and 3.2 Å (*d*). In the apo sample, density for Trp142 was also weaker when the Rieske domain was in the intermediate position (Supplementary Fig. S7).

Map density for the Rieske domain was less good at positions in which the cluster-bearing tip did not interact closely with either cyt *b* or cyt *c*_1_. However, sample-dependent differences in the position of the Rieske domain (Fig. 4 ▸) as well as changes to cyt *b* that appeared to correlate with the position of the Rieske domain (Fig. 5 ▸) were observed in some samples.

Rigid-body fitting of the Rieske domain in the *c* position showed that it was closer to heme *c*_1_ in samples with fully or partially reduced high-potential chains (Apo, +NaAsc, +NaAsc+DQ and +DQH2) compared with fully oxidized samples (+FCN and +DQ) (Figs. 2 ▸ and 4 ▸). Under these conditions, the cluster-bearing tip of the Rieske domain was best resolved when ascorbate was added. His190 was within 3.4 Å of the propionic acid group of heme *c*_1_. In the *c* position of partially reduced CIII_2_ (apo) His190 was within 3.8 Å of the propionic acid group, whereas it did not approach heme *c*_1_ closer than ∼8 Å in the fully oxidized complex (+FCN). At a lower threshold, an undefined, rod-shaped density was visible in the Q_o_ site, which is accessible to solvent with the Rieske domain in the *c* position.

Interestingly, in samples without added reductant or oxidant, and especially in the presence of antimycin A, map densities for the *cd*_1_-helix and *ef*-loop of cyt *b* were weaker and modelling was more difficult when the Rieske domain was in the intermediate position (Fig. 5 ▸). In particular, density for Trp142 of the *cd*_1_-helix and Ile269 of the *ef*-loop deviated from that observed in maps of the same sample with the Rieske domain in the *b* position. In addition, densities for Val146, Ile147, Cys148 and Asn149 of the *cd*_1_-helix were ambiguous. In maps of fully reduced or oxidized samples this area of cyt *b* was well-resolved irrespective of the position of the Rieske domain. Trp142 appeared to be out of its consensus position underneath the top of the *ef*-loop and in a position towards the Q_o_ site. In this position, Trp142 would partially overlap with Ile269. In the apo sample without antimycin A, density for Trp142 appeared to be very weak with partial occupancy in both positions (Supplementary Fig. S7).

### 3.4. Reconstructions with both Rieske domains in the dimer

**Figure 6:**
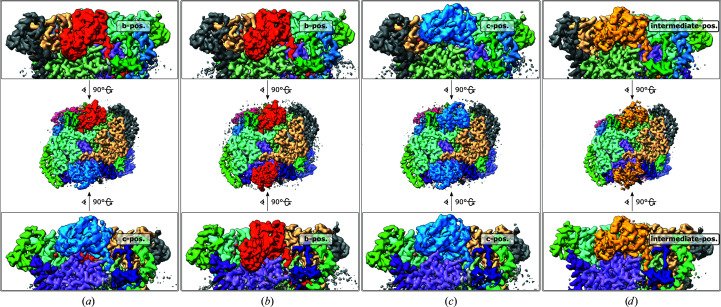
Typical maps from the +FCN sample with both Rieske domains in the CIII dimer. Rieske domains are coloured red, yellow and blue for the *b*, intermediate and *c* positions. (*a*) Refinement of particle pairs with one protomer in the *b* position and one in the *c* position (30 714 particles, 2.7 Å resolution, *C*1). (*b*, *c*, *d*) Maps from the refinement of particle pairs with both protomers in the *b* position (6727 particles, 3.0 Å resolution, *C*2), *c* position (11 987 particles, 2.8 Å resolution, *C*2) and intermediate position (9819 particles, 2.8 Å resolution, *C*2). All possible permutations of positions were observed.

For the reconstruction of both Rieske domains in the dimer, the six classes of symmetry-expanded particles were further split into subsets after focused 3D classification. Every particle was sorted according to the combination of the two respective classes that were assigned to the symmetry-expanded pair during focused 3D classification. Local 3D refinements of these particle subsets yielded maps of CIII_2_ with improved density for both ISP-ED protomers (Fig. 6 ▸). There are 21 combinations of six classes possible in CIII_2_. Excluding classes with blurred Rieske domains and combining very similar classes (as judged by the proximity of the positions of the fitted [2Fe–2S] cluster to heme *b*_L_ and heme *c*_1_ in Fig. 2 ▸), this still left up to 15 unique maps per sample, as was the case with +NaAsc and +DQ+NaAsc with ∼2000–25 000 particles per class and overall resolutions of 7.5–3.4 Å (without masking). We found both conformationally symmetric (*C*2) and asymmetric (*C*1) dimers, indicating that the two Rieske domains in the dimer move independently.

## 4. Discussion

We report multiple high-resolution cryo-EM structures of CIII_2_ from *Y. lipolytica* with a focus on the small, ∼14 kDa iron–sulfur cluster-binding domain of the Rieske protein subunit. The Rieske domain plays a central role in electron transfer through CIII_2_. As expected, the Rieske domain was mobile and thus poorly resolved, except when the P_f_-type inhibitor atovaquone was added to arrest the domain at the surface of cyt *b* (Birth *et al.*, 2014 ▸). Cryo-EM images of CIII_2_ particles were classified according to the position of the Rieske domain in the dimer maps, thus reducing conformational heterogeneity in subsequent refinement steps. Due to the small size and weak signal of the Rieske domain separation was not perfect but, in the more restrained *b* positions in particular, details of the Rieske domain and density for the [2Fe–2S] cluster improved substantially. The higher resolution of the Rieske domain indicates that it has less conformational freedom in the *b* position than in the intermediate and *c* positions. The Rieske domain covered a range of positions between the *b* and the *c* position. We found a systematic effect of the redox state of the high-potential chain and of added inhibitors or substrate analogues on the position of the Rieske domain in the complex, with direct consequences for electron transfer. As expected, the distances between the [2Fe–2S] cluster and the Q_o_ site or heme *c*_1_ are within 14 Å for the *b* and *c* positions, respectively, at which electron tunnelling between redox cofactors is not rate-limiting for CIII_2_ turnover (Zhang *et al.*, 1998 ▸; Iwata *et al.*, 1998 ▸; Page *et al.*, 1999 ▸).

Consistent with previous EPR experiments (Brugna *et al.*, 2000 ▸), the Rieske domain was found in a position closer to cyt *b* when the high-potential chain was reduced by sodium ascorbate compared with when it was fully or partially oxidized. The interaction between cyt *b* and the Rieske domain of the reduced high-potential chain appeared to be weaker when there was an excess of DQH_2_, as the Rieske domain was confined to a more distal position. This highlights the role of the hydrogen bond between the cluster-ligating His190 of the Rieske domain and the Q_o_-site ligand in positioning the Rieske domain on the surface of cyt *b*. In CIII_2_ with a reduced high-potential chain there was also a clear increase in density for bound quinone in Q_o_ when DQ was added. The hydrogen bond can form in the enzyme–product complex when the [2Fe–2S] cluster is reduced and Q is bound or when the cluster is oxidized and QH_2_ is bound, forming the reactive enzyme–substrate complex (Crofts *et al.*, 1999 ▸). Additional stabilization of the *b* position may arise through interaction of the backbone carbonyl of Cys189 or the cluster-ligating His190 with Tyr279 of cyt *b*. The His190–Tyr279 hydrogen bond is also seen with P_f_-type inhibitors such as famoxadone that do not form a direct hydrogen bond to His190 but still arrest the Rieske domain in the *b* position (Gao *et al.*, 2002 ▸; Berry & Huang, 2011 ▸). However, widening of the docking crater on cyt *b*, as seen in P_f_-type inhibitor-bound structures (Esser *et al.*, 2006 ▸; Birth *et al.*, 2014 ▸), was not observed in the *b* positions of apo CIII_2_ or of CIII_2_ when a substrate analogue was added. Therefore, it remains unclear whether cyt *b* acts as a catalytic switch with native substrate, in the same way as Q_o_-site inhibitors.

Despite the closer interaction of the Rieske domain with cyt *b*, no shift in the equilibrium of positions in ascorbate-reduced samples was observed. Together with the weak density in the hinge region between the Rieske and transmembrane domains in these samples, this may indicate that the close interaction of the cluster-bearing tip with cyt *b* loosens the helical secondary structure of the hinge only transiently (Darrouzet *et al.*, 2000 ▸). This would be consistent with a spring-loaded mechanism that prevents prolonged association of cyt *b* and the Rieske domain (Crofts *et al.*, 2002 ▸; Sarewicz *et al.*, 2021 ▸; Berry *et al.*, 2013 ▸).

The densities of the *cd*_1_-helix and the *ef*-loop were weaker and more difficult to model when the Rieske domain was in the intermediate position in CIII_2_ samples without added oxidant or reductant, indicating increased flexibility and heterogeneity. The addition of antimycin A reduced the resolution of these regions, whereas both the *cd*_1_-helix and the *ef*-loop were well-resolved in the intermediate positions of CIII_2_ oxidized with ferricyanide or reduced with ascorbate or DQH_2_. If Trp142 of the *cd*_1_-helix moves out from underneath the *ef*-loop this is likely to increase the mobility of this loop and thereby reduce the energetic barrier to the transition of the Rieske domain between the *b* and *c* positions. This might explain the increased mobility of the Rieske domain upon the addition of antimycin A (Valkova-Valchanova *et al.*, 2000 ▸; Cooley *et al.*, 2005 ▸; Sarewicz *et al.*, 2009 ▸). To our knowledge, Trp142 has never been resolved in this position or in crystal structures with bound antimycin A (Gao *et al.*, 2003 ▸). However, in the crystal structure the Rieske domain was modelled in the *b* position, where we also observed the majority of density for Trp142 in its common position. In our structures and in those previously published, no changes upon the binding of antimycin A were apparent that could explain long-range communication between the Q_i_ and Q_o_ sites. Also, increased heterogeneity was not observed in cyt *b* when antimycin A was added in combination with atovaquone or decylubiquinol. Therefore, further experimental evidence and higher resolution maps would be required to validate this observation. Trp142 is highly conserved (Esposti *et al.*, 1993 ▸) and is known to be important for electron transfer through the high-potential chain (Lemesle-Meunier *et al.*, 1993 ▸; Bruel *et al.*, 1995 ▸).

When the high-potential chain was partially or fully reduced either by sodium ascorbate or an excess of DQH_2_, *c* positions closer to heme *c*_1_ were observed. The resolution of *c* positions was limited, but in the rigid-body fitted Rieske domain His190 came within hydrogen-bonding distance of the heme *c*_1_ propionate, as in previous crystal structures (Iwata *et al.*, 1998 ▸).

It was also possible to classify particle images according to the positions of both Rieske domains in the dimeric complex. We found both conformationally symmetric and asymmetric dimers, as reported by others (Steimle *et al.*, 2021 ▸; Di Trani *et al.*, 2022 ▸). The two Rieske domains within the CIII dimer therefore appear to move independently of one another. However, a coordinated movement may be imposed in the context of higher-order respiratory complexes (Letts *et al.*, 2016 ▸, 2019 ▸; Sousa *et al.*, 2016 ▸; Moe *et al.*, 2021 ▸).

The structures presented in this paper provide evidence that the redox state of the high-potential chain affects the interaction of the Rieske protein with cyt *b* and cyt *c*_1_. The reduced state of the high-potential chain favours closer interaction with both cyt *b* and and cyt *c*_1_ without apparent shifts in the equilibrium of positions, enabling rapid electron transfer between the cofactors of the high-potential chain. Under conditions that favour the enzyme–product complex we observe better density for a quinone substrate in the Q_o_ site, which has been difficult using crystallographic studies. The heterogeneity of the *cd*_1_-helix and *ef*-loop increased upon the binding of antimycin A, which may affect the dynamics of the Rieske domain.

## Supplementary Material

PDB reference: CIII_2_, consensus refinement, 8ab6

PDB reference: with atovaquone and antimycin A bound, 8ab7

PDB reference: ascorbate-reduced with decylubiquinone, Rieske domain in *b* position, 8ab8

PDB reference: ascorbate-reduced, Rieske domain in *b* position, 8ab9

PDB reference: ascorbate-reduced, Rieske domain in intermediate position, 8aba

PDB reference: ascorbate-reduced, Rieske domain in *c* position, 8abb

PDB reference: ferricyanide-oxidized, Rieske domain in *b* position, 8abe

PDB reference: ferricyanide-oxidized, Rieske domain in intermediate position, 8abf

PDB reference: ferricyanide-oxidized, Rieske domain in *c* position, 8abg

PDB reference: with antimycin A bound, Rieske domain in *b* position, 8abh

PDB reference: with antimycin A bound, Rieske domain in intermediate position, 8abi

PDB reference: with antimycin A bound, Rieske domain in *c* position, 8abj

PDB reference: with decylubiquinol, Rieske domain in *b* position, 8abk

PDB reference: with decylubiquinol and antimycin A, consensus refinement, 8abl

PDB reference: Rieske domain in *b* position, 8abm

PDB reference: Rieske domain in intermediate position, 8ac3

PDB reference: Rieske domain in *c* position, 8ac4

PDB reference: with decylubiquinone, Rieske domain in *b* position, 8ac5

Supplementary table and figures. DOI: 10.1107/S2052252522010570/rq5008sup1.pdf
