# Supplementary material for: Analysis of the conformational heterogeneity of the Rieske iron–sulfur protein in complex III2 by cryo-EM
Source: IUCrJ. 2023 Jan 1;10(Pt 1):27–37. doi: 10.1107/S2052252522010570 (PMC9812224; doi:10.1107/S2052252522010570)
Supplement: Supplementary file 1 [file m-10-00027-sup1.pdf]

IUCrJ

Volume 10 (2023)

Supporting information for article:

Analysis of the conformational heterogeneity of the Rieske iron–sulfur protein in $CIII_2$ by cryo-EM

Jan-Philip Wieferig and Werner Kühlbrandt

Table S1 Cryo-EM data collection, refinement and validation statistics

	consens. refine. combined datasets,	b-pos, DQ, ascorbate	b-pos, ascorbate	b-pos, ferri-cyanide	b-pos, apo	b-pos, antimycin	b-pos, DQH_2	consens. refine., DQH_2 , antimycin	Ato-vaquone, antimycin
Magnification	105,000	105,000	105,000	105,000	105,000	105,000	105,000	105,000	105,000
Voltage (kV)	300	300	300	300	300	300	300	300	300
Electron exp-osure (e-/Å^2)	55	55	55	55	55	55	55	55	55
Defocus range (µm)	-0.9 to -2.2	-0.9 to -2.2	-0.9 to -2.2	-0.9 to -2.2	-0.9 to -2.2	-0.9 to -2.2	-0.9 to -2.2	-0.9 to -2.2	-0.9 to -2.2
Pixel size (Å)	0.83	0.83	0.83	0.83	0.83	0.83	0.83	0.83	0.83
Symmetry	C2	C1	C1	C1	C1	C1	C1	C2	C2
Particle images	1,419,666	57,703	33,680	112,357	138,795	45,247	69,259	225,162	35,394
Map resolution (Å) (0.143 cut-off)	2.0	2.6	3.3	2.3	2.8	3.0	2.5	2.1	3.3
Map resolution (Å) (0.5 cut-off)	2.2	3.1	3.7	2.8	3.2	3.8	3.0	2.5	3.7
**Refinement**									
Protein residues	1,939	1,939	1,939	1,939	1,939	1,939	1,939	1,939	1,939
Ligands	HEC: 2, HEM: 4, CDL: 10, LMT: 4, PC1: 4, PTY: 4, XP4: 2, H_2O: 1,326	HEC: 2, HEM: 4, DCQ: 1, FES: 1, CDL: 10, LMT: 4, PC1: 4, PTY: 4, XP4: 2,	HEC: 2, HEM: 4, FES: 1, CDL: 10, LMT: 4, PC1: 4, PTY: 4, XP4: 2,	HEC: 2, HEM: 4, FES: 1, CDL: 10, LMT: 4, PC1: 4, PTY: 4, XP4: 2,	HEC: 2, HEM: 4, FES: 1, CDL: 10, LMT: 4, PC1: 4, PTY: 4, XP4: 2,	HEC: 2, HEM: 4, FES: 1, AWB: 2, CDL: 10, LMT: 4, PC1: 4, PTY: 4, XP4: 2,	HEC: 2, HEM: 4, FES: 1, DCQ: 2, CDL: 10, LMT: 4, PC1: 4, PTY: 4, XP4: 2,	HEC: 2, HEM: 4, AWB: 2, CDL: 10, LMT: 4, PC1: 4, PTY: 4, XP4: 2,	HEC: 2, HEM: 4, FES: 2, AOQ: 2, AWB: 2, CDL: 10, LMT: 4, PC1: 4, PTY: 4, XP4: 2,
RMSD bond length (Å^2)	0.006	0.006	0.013	0.006	0.007	0.009	0.006	0.006	0.010
RMSD bond angles (°)	1.531	1.541	1.714	1.726	1.690	1.791	1.667	1.685	1.919
Molprobity	1.38	1.63	1.75	1.61	1.69	1.80	1.63	1.63	2.13
Clashscore	3	3	4	3	3	4	4	3	7
Sidechain outliers (%)	2.0	3.3	3.5	3.3	3.5	3.5	3.2	4.6	5.1
Ramachandran favoured (%)	97.65	97.47	97.45	97.52	97.40	97.37	97.55	97.71	96.96
Ramachandran outlier (%)	0.0	0.0	0.1	0.0	0.1	0.1	0.0	0.1	0.1

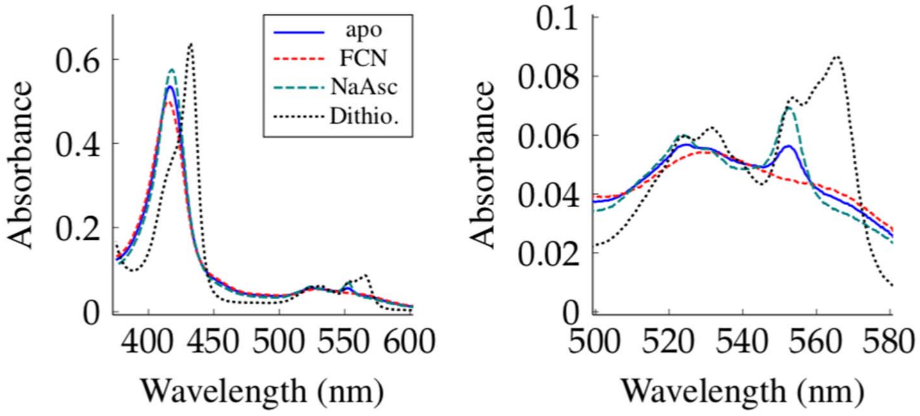

(A)

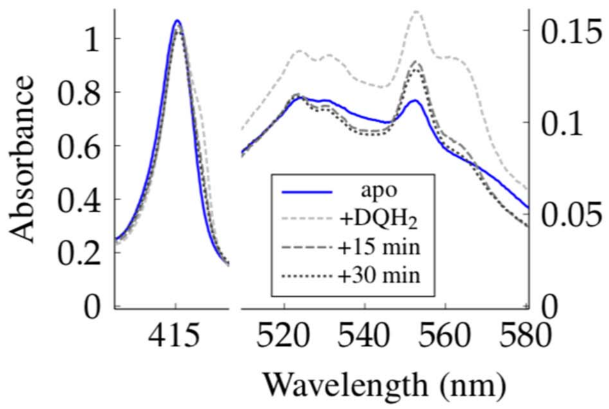

(B)

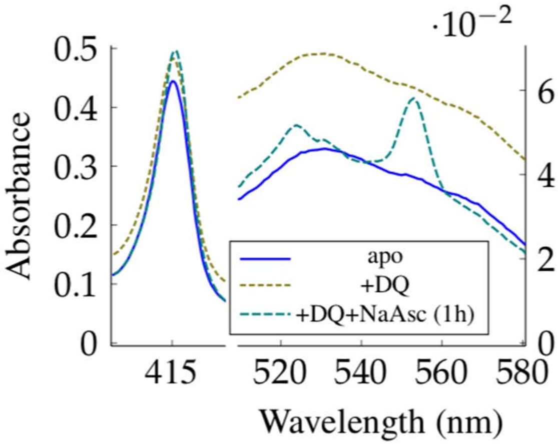

(C)

Figure S1 UV/Vis-spectra of $CIII_2$ after purification and after addition of ferricyanide (+FCN), ascorbate (+NaAsc) or dithionite (a), addition of the reduced substrate analogue decylubiquinol (+DQH_2) (b) or decylubiquinone (+DQ) with and without ascorbate (c). Reduction of hemes c_1 led to a shift of the Soret peak from 415 nm to 417-418 nm, increased absorbance in the beta-band at 524 nm and a peak in absorbance at 552 nm. Reduction of b hemes is indicated by a shift of the Soret peak to 432 nm and overlapping absorbance peaks of heme b_L and heme b_H at 532, 560, 558 and 566 nm. Reduced b hemes re-oxidized quickly under aerobic conditions. Poor solubility of substrate analogue resulted in initial turbidity sample.

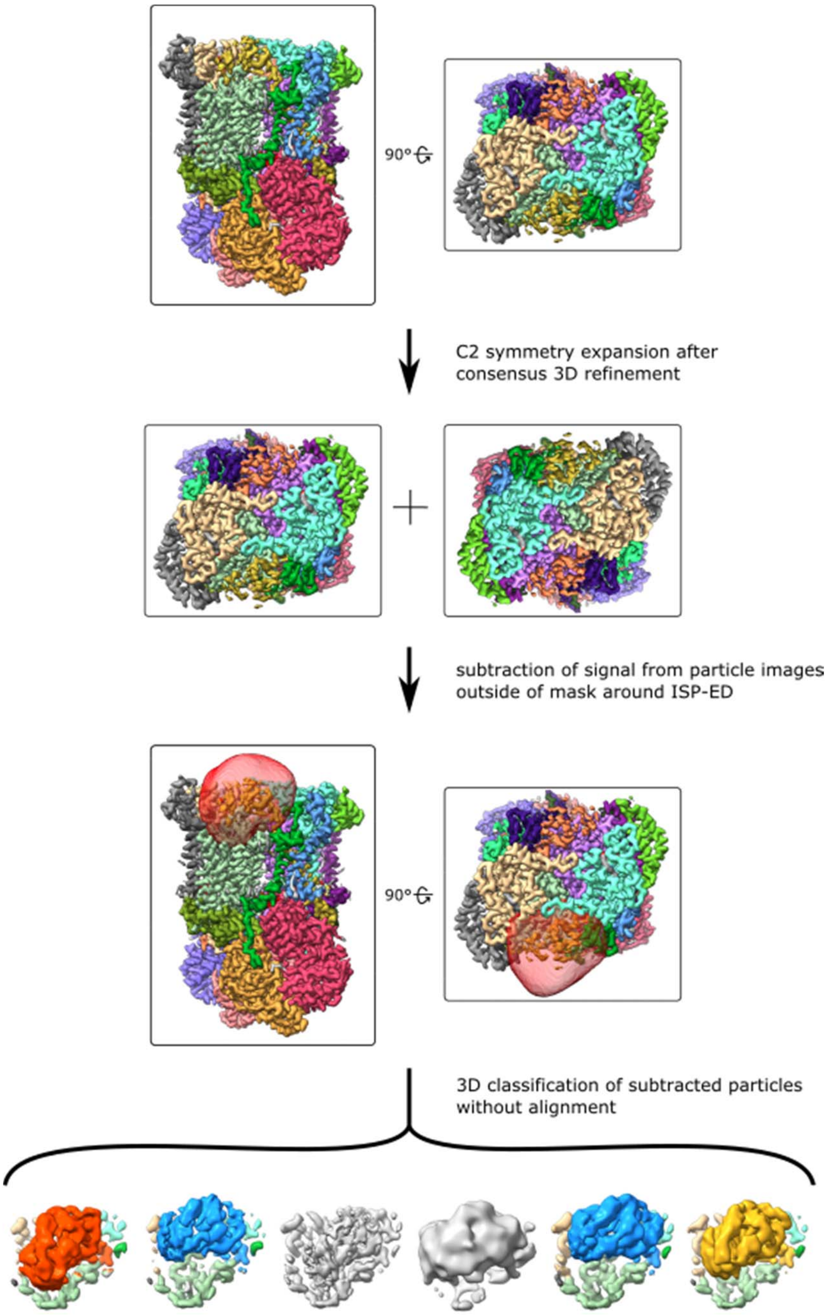

Figure S2 Workflow for the focused 3D-classifications after symmetry expansion and subtraction of density outside a mask surrounding the Rieske domain.

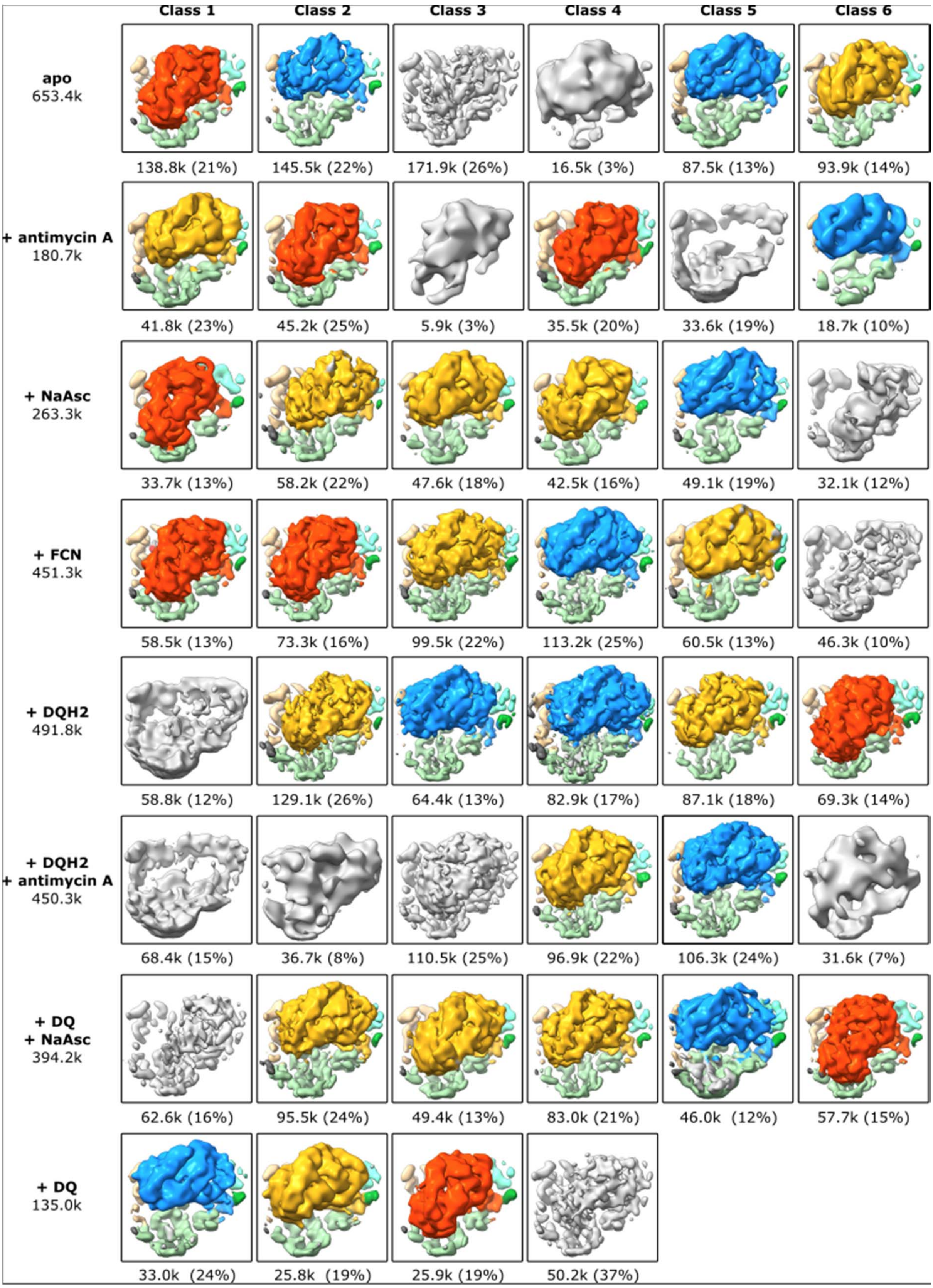

Figure S3 Results of the focused 3D-classifications of all samples show b- (red), intermediate (yellow) and c-positions (blue) of the Rieske domain within each dataset. Particle numbers refer to C2-symmetry-expanded dataset (2x).

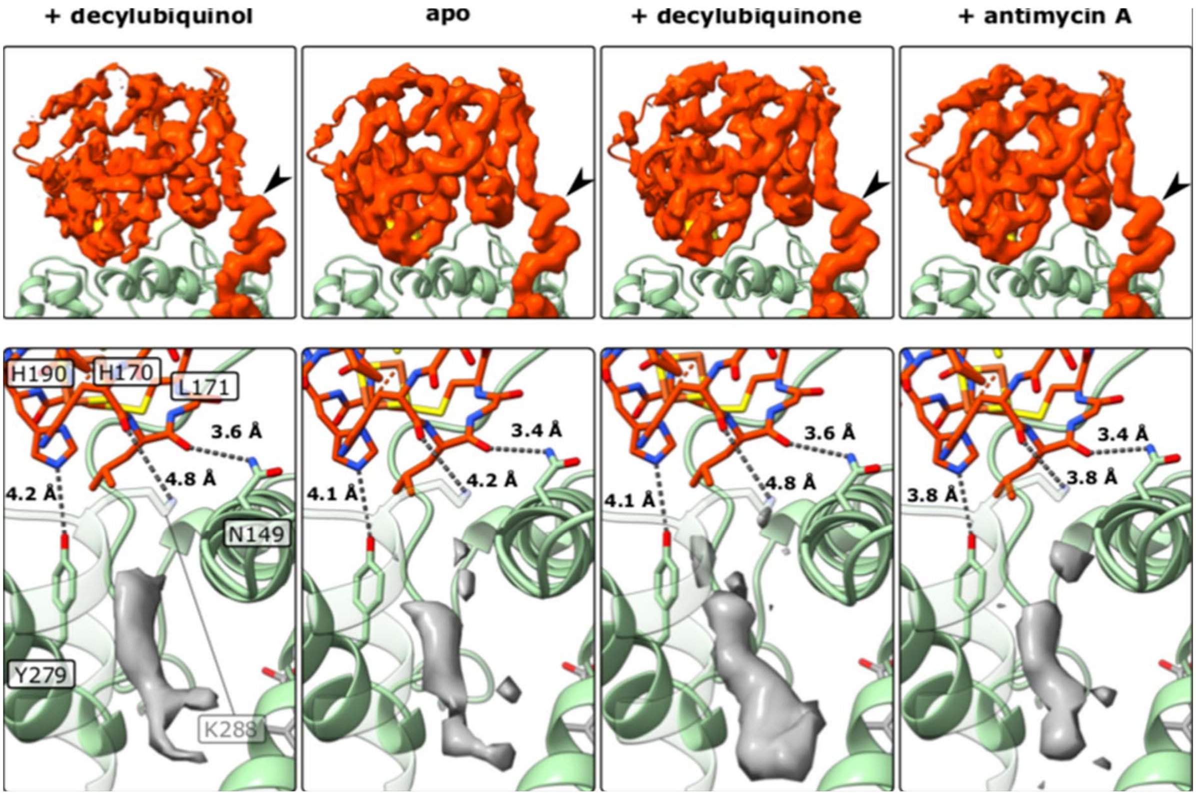

Figure S4 b-positions not shown in **fig. 3**. Density of the experimental map in the region of the Rieske domain (red) is shown in the top panels. The lower panels show details of the Q_o site showing poorly-defined, low-threshold density in the substrate-binding cavity.

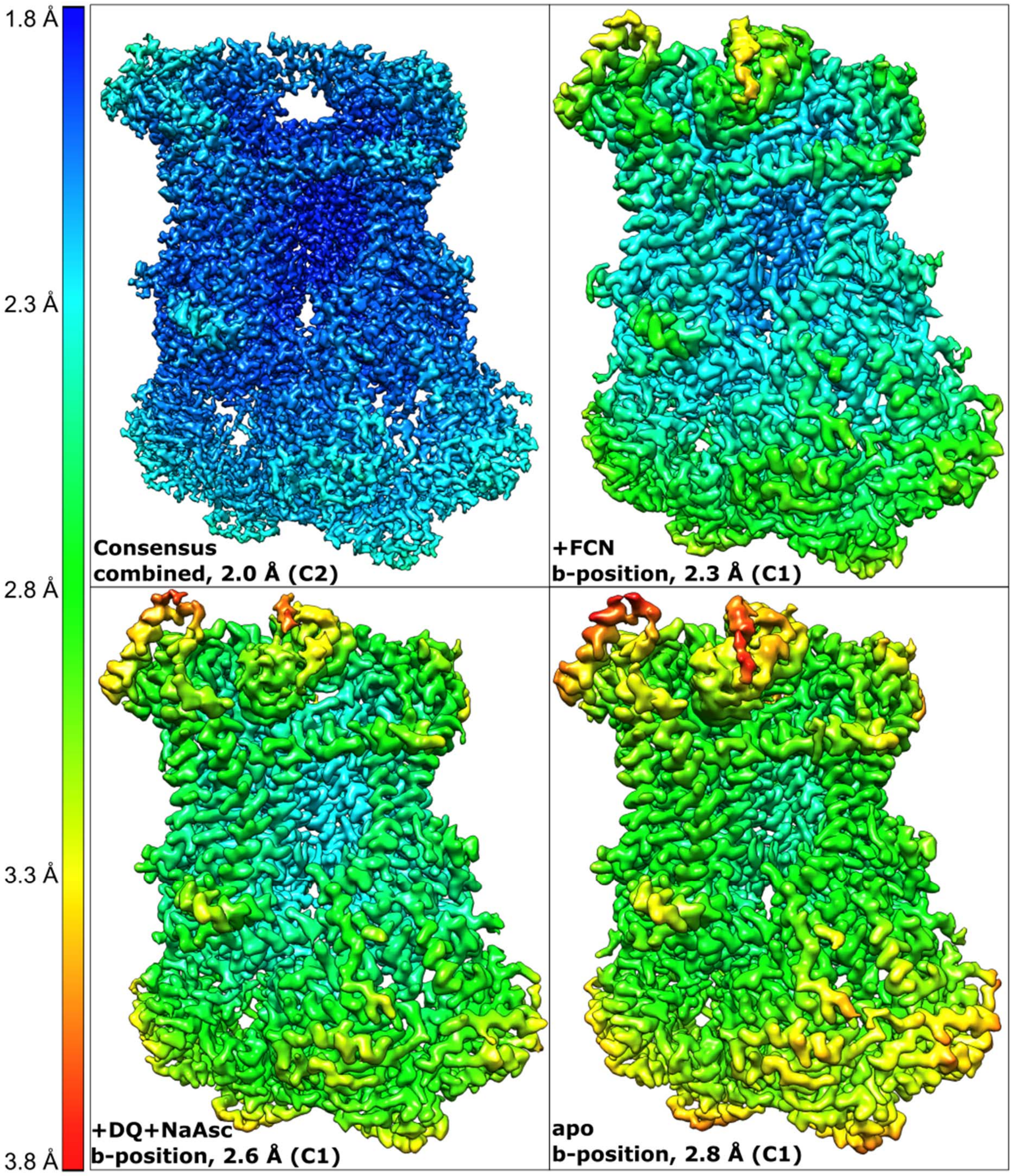

Figure S5 CryoEM density maps of $CIII_2$ coloured by local resolution after consensus refinement of combined datasets without atovaquone (Rieske domains not resolved), and with one Rieske domain in b-position of maps shown in fig. 3 and supplementary fig. 4.

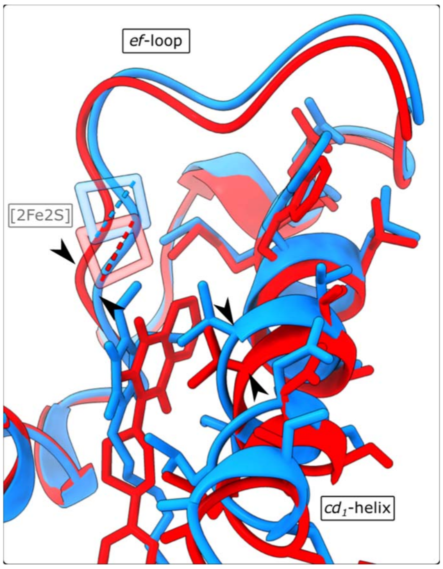

Figure S6 Superposition of cyt b of samples with atovaquone (red) and ascorbate-reduced with decylubiquinone added (blue). Atovaquone binding induces an expansion of the Q_o site (Esser et al., 2006, Birth et al., 2014) by displacement of the cd_1-helix and ef-loop (arrows). This allows tighter docking of the Rieske domain to cyt b compared to non-inhibitor bound $CIII_2$ as indicated by the positions of the [2Fe2S] clusters (opaque).

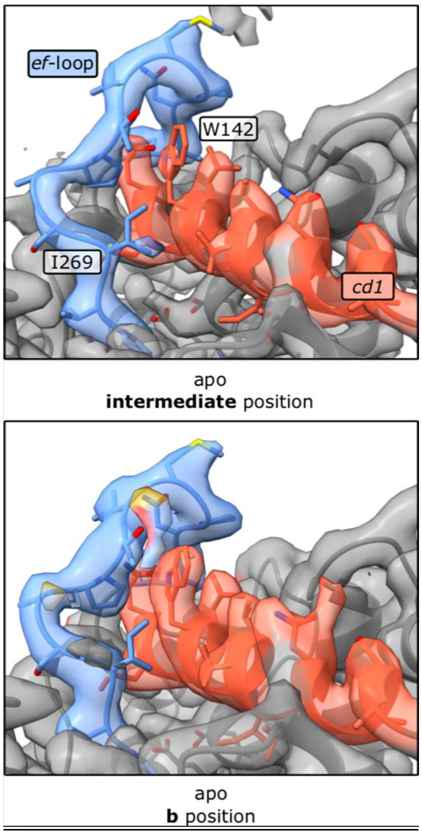

Figure S7 Trp142 of the cd_1-helix is poorly resolved in the apo sample, when the Rieske domain is in the intermediate position.
